# Environmental and Health Impacts of Crop Residue Burning: Scope of Sustainable Crop Residue Management Practices

**DOI:** 10.3390/ijerph19084753

**Published:** 2022-04-14

**Authors:** Muhammad Haseeb Raza, Muhammad Abid, Muhammad Faisal, Tingwu Yan, Shoaib Akhtar, K. M. Mehedi Adnan

**Affiliations:** 1 Department of Agribusiness and Applied Economics, MNS University of Agriculture, Multan 60000, Pakistan; haseeb.raza@mnsuam.edu.pk; 2 Deutsche Gesellschaft Für Internationale Zusammenarbeit (GIZ) Level 2, Islamabad 45550, Pakistan; abiduaf@gmail.com; 3 Department of Economics, University of Lahore, Sargodha Campus, Sargodha 40100, Pakistan; faisalgurmani@gmail.com; 4 College of Economics & Management, Huazhong Agricultural University, Wuhan 430070, China; 5 Hubei Rural Development Research Centre, Wuhan 430070, China; 6 Centre of Excellence for Olive Research & Training (CEFORT), Barani Agriculture Research Institute (BARI), Chakwal 48800, Pakistan; shoaibakhtar1999@gmail.com; 7 Department of Agricultural Finance & Banking, Sylhet Agricultural University, Sylhet 3100, Bangladesh; mehedi_adnan@yahoo.com

**Keywords:** health cost, sustainable crop residue management, propensity score matching, environmental benefits, Pakistan

## Abstract

The burning of crop residue in the open field has become a significant concern for climate change mitigation efforts worldwide. This practice has led to air quality impairment, smog, haze, heat waves, and different health problems. These could be avoided by adopting sustainable crop residue management practices (SCRMPs) and enabling farmers to engage in SCRMPs. Assessing the health effects at the household level is critical for understanding this problem and finding a solution. Using the primary dataset of 420 farmers from Punjab, Pakistan, we estimated the incurred impacts and costs of crop residue burning. We calculated the health and environmental benefits associated with adopting SCRMPs by comparing the two groups of farmers (adopters and non-adopters). Furthermore, we used a propensity score matching technique to measure the causal impact of SCRMPs adoption on health costs. The findings showed that a surprisingly large number of farmers are all aware of the adverse effects of residue burning, and many do not burn crop residues and instead use SCRMPs. This study found that households with chronic and non-chronic diseases become acute, and the severity increases during the burning period. They spend USD 13.37 to USD 8.79 on chronic and non-chronic diseases during the burning season, respectively. Consequently, the use of SCRMPs has a positive effect on healthcare costs. Our study findings highlight the meaningful implications for developing a new policy to promote the sustainable utilization of crop residues and enhance their adoption in Pakistan.

## 1. Introduction

Economic development and rapid population growth have put intense pressure on the agriculture sector to fulfill rising industrial and food demands [1,2,3]. As a result, intensive agriculture with high use of inputs and the introduction of modern technologies has developed rapidly in recent decades [4,5,6]. The use of synthetic fertilizers and pesticides, as well as the extension of cultivated land, exploitation of natural resources, and burning of crop residues to clear fields for preparations of the new crops have all resulted in a slew of environmental issues [7,8,9,10], including water contamination, air quality impairment, the emission of greenhouse gases (GHG) and soil degradation [11,12,13]. These problems are not only impairing environmental sustainability but also pose a severe threat to human health.

Increasing greenhouse gas emissions due to rapid industrialization and urbanization in developing countries have contributed to the changing climate [14]. Furthermore, crop residue burning has proven to be a non-point source of greenhouse gas emissions in many agrarian economies, including Pakistan [12,15]. Over the past few decades, Pakistan’s agriculture sector has been under tremendous pressure to meet the food needs of a population of more than 220 million, rising at rate of nearly 2% each year [16]. This immense pressure has led to the intensification of agriculture and multiple cropping across the country. The current cropping intensity (159%) also shows intense competition between different crops due to the harvesting and sowing seasons overlapping.

Consequently, farmers often engaged in unsustainable practices such as burning crop residues to clear fields for the following crop [17,18]. In recent years, massive crop residue burning has resulted in air quality deterioration, smog, haze, heat waves, and different health issues [19,20,21,22]. In severe circumstances, it has led to an emergency situation with several deaths, thousands of hospital admissions, temporary closure of airports, highways, and schools [22,23,24,25], as well as significant economic losses [26,27].

Given the growing adverse impacts of crop residue burning, adopting sustainable and environmentally friendly residue management practices is required to safeguard the environment and people’s health [28,29,30]. Numerous studies have reported several environmentally friendly crop residue management practices [31,32,33] that provide additional economic benefits through generating income and reducing healthcare expenses. However, the adoption rate is quite low, even though the positive impacts of sustainable crop residue management practices (SCRMPs) are widely studied, understood, and implementable at the local level [34,35,36,37]. The low adoption rate may be due to a lack of information about the benefits of such strategies, particularly their environmental and health implications.

Limited research on the positive aspects of SCRMPs could be another reason for its low adoption at the farm level. Few studies have documented crop residue burning, focusing on the negative impacts or the expense of managing crop residues at the farm level [38,39]. For instance, Riaz and Hamid (2018) [22] highlighted that crop residue burning is now restricted to a few places in Punjab province, but if it continues, it will spread across all the plain areas of Pakistan. Mendoza (2015) [40] also suggested that recycling crop residues in the landscape of the Philippines would be an excellent approach to reducing the country’s reliance on imported fertilizers while simultaneously helping to combat climate change. Moreover, little research has been conducted on exploring the nexuses between the positive aspects of adopting SCRMPs and their impact on human health [41,42]. Therefore, further research is needed to explore the benefits of SCRMPs and the underlying factors and constraints that prevail at the local level.

Given the aforementioned research gap, this study will provide better insight into farmers’ adoption of SCRMPs and their impact on human health. Precisely, this paper addresses four important research questions: First, how do farmers respond to SCRMPs? Second, how does the adoption of SCRMPs differ among farmers? Third, what factors sway farmers’ decisions to implement SCRMPs? Fourth, what impact does the adoption of SCRMPs have on human health costs?

## 2. Materials and Methods

### 2.1. Study Area

**Figure 1:**
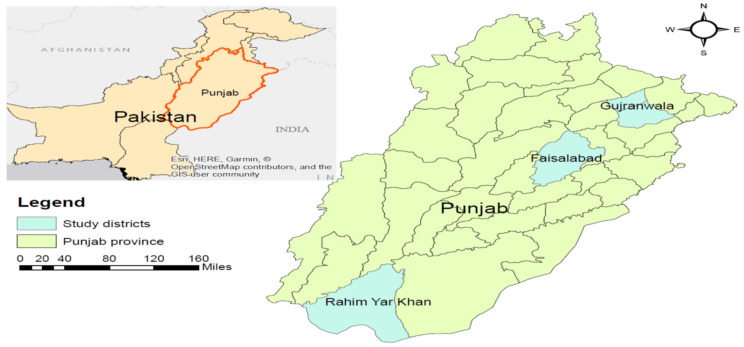
Research area.

Punjab is the primary focus of this research since it produces 74 percent of the country’s cereal and contributes 53 percent of the country’s agricultural GDP. The major crops cultivated here are wheat, rice, sugarcane, maize, cotton and pulses [43,44]. In Punjab, five agro-climatic zones, namely wheat–rice zone, cotton–wheat zone, mixed cropping zone, barani (arid) zone, and low crop intensity zone, exist [45,46]. The rice-wheat cropping zone is very important, as rice is the most important crop in the kharif season (starts from June and ends in October). As reported by different studies, the majority of crop residue is managed through open field burning, and the remainder is utilized for domestic cooking [47,48]. Moreover, in the mixed cropping zone rice-wheat sugarcane is cultivated and the majority of the residue is managed through burning [39,49]. Consequently, this province is also responsible for widespread crop residue burning, which has resulted in severe health and environmental consequences. We compute the variation in different crop residue practices. This study was mainly conducted in three districts selected from the rice–wheat zone (Gujranwala district), mixed cropping zone (Faisalabad district), and cotton–wheat zone (Rahim Yar Khan). A map of the research area and selected districts is shown in Figure 1.

### 2.2. Data Source

**Figure 2:**
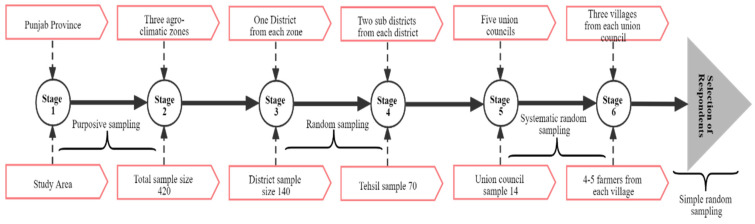
Sampling strategy.

For this study, cross-section data were collected from selected districts using structured questionnaires from February to March 2018. A multi-stage sampling technique (District→Tehsil→Union council→Villages→Respondents) was used to select approximately 420 farmers for face-to-face interviews (Figure 2). The purposive sampling technique was used in the selection of Punjab province (first stage) and representative agro-climatic zones (second stage). Random sampling was used in the selection of representative districts (third stage) from each agro-climatic zone. From each district, two sub-districts (tehsils) (fourth stage) and 15 villages (fifth stage) were selected through a systematic random sampling technique. Finally, a simple random sampling technique was used in the selection of 4–5 farmers (sixth stage) from each village. In short, two tehsils were selected from each district, 5 union council from each tehsil, 3 villages from each union council, and 4–5 farmers from each village. A representative sample was determined by considering various aspects such as demographic, socio-economic, and geography profiles of the households in the study areas of three agro-ecological zones. Various indicators used in the current study were primarily based on the authors’ own understanding of the study location, along with following peers and the published literature [50,51]. Respondents’ responses were verified from key informant interviews before the final field observation. For this purpose, a list of farmers was acquired from the respective agriculture department. For the data collection, enumerators were hired from an agriculture university and trained. One interview took, on average, 30–40 min due to the low literacy rate of the respondents and to ensure the precision of the collected data. A total of 25 farmers refused or did not participate in the survey due to a lack of understanding. A protocol was followed, ensuring no identifying information was available except the village name and serial number of the questionnaire. Farmers were questioned to gain socio-economic information, demographic characteristics, and information on diseases, costs associated with health problems, number of working days lost, and adoption of SCRMPs to safeguard the environment and human health.

### 2.3. Conceptual and Analytical Framework

**Figure 3:**
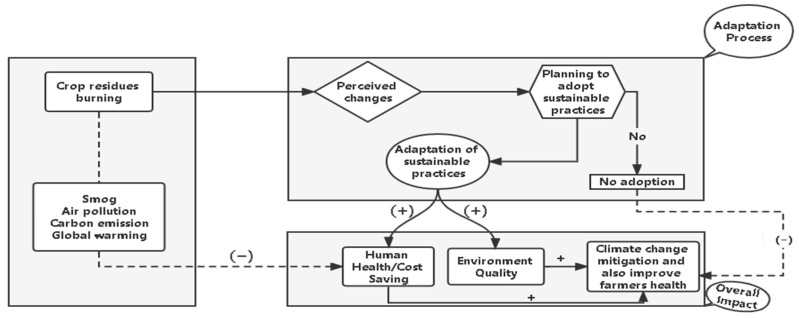
Conceptual framework.

Sustainable agriculture production is inevitable due to rapid urbanization, the mass spread of modern technologies, and the extensive use of inputs in order to ensure economic growth. This immense pressure has led to the intensification of agriculture, with farmers often engaging in unsustainable practices, resulting in a slew of environmental pollution. It is too simplistic to assume that unprecedented agriculture pollution would minimize by promoting sustainable/eco-friendly agricultural practices [52,53]. So, it is necessary to understand the concept of SCRMPs in order to ensure the best outcome from any promotional or awareness campaigns. The conceptual framework of this study consists of three parts, as shown in Figure 3: environmental and health vulnerabilities, the adoption mechanism, and overall adoption impact. The relationship between environmental vulnerabilities, the adoption process, and human health is depicted using straight and dotted lines. The negative consequences such as increased healthcare costs and poor environmental quality are depicted in particular by dotted lines.

On the other hand, the straight lines show the positive impacts of SCRMPs’ adoption on the environment and improve household’s health conditions in terms of welfare. Interestingly, the authors of [22,54] reported many environmental and health vulnerabilities from the big cities of Punjab, Pakistan, and linked these with residue burning. Additionally, extreme temperatures, heatwaves, and hazardous smog have been observed in Pakistan’s south to central regions in recent years [24]. It can potentially harm farmers’ health and production and their overall well-being. Poor health can have a negative impact on labor productivity and thereby affect farmers’ well-being. However, if farmers adopt SCRMPs, it will improve their health and positively impact productivity. In short, continual residue burning would result in low crop production and detrimental effect on the environment and health. This study aims to test the interactions, as explained above, and hypotheses.

#### 2.3.1. Adoption Decision

In the context of the current investigation, the adoption of SCRMPs is defined as a preventive measure to offset losses caused by poor air quality, smog, and pollution. A farmer is considered an adopter if he does not burn crop residues and instead employs sustainable residue management measures; if he burns the residues, he is regarded as a non-adopter.

Following Kato et al. (2011) [55], we used a random utility framework to represent farmer adaptation decisions. Here, we assumed that *i*th farmer would choose to adopt sustainable residue management practices only if he expected less expenditure on health issues and is curious about environmental pollution or anticipated benefits from adoption. The adoption benefits may reduce the monetary losses and improve the local environment and health of farm households. This disparity in benefits can be expressed using a latent variable: $\left(U_{i}^{*}\right)$:

(1) $$\begin{array}{l} U_{i}^{*}=\beta Z_{ik}+\mu_{i} \end{array}$$

where $Z_{ik}$ is the *k* explanatory variables’ vector, β is the logistic regression coefficients vector and $\mu_{i}$ is the error term. As the latent variable $\left(U_{i}^{*}\right)$ is unobservable, we have only:

(2) $$U_{i}=\{\begin{array}{c} 1,\;U_{i}^{*}>0\\ 0,\;U_{i}^{*}\leq 0 \end{array}$$

where $U_{i}$ indicates that the *i*th farmer will adopt sustainable residue management practices $\left(U_{i}=1\right)$ only if the cost of medication reduces from stopping the burning of residue $(U_{i}^{*}$ > 1). In contrast, *i*th farmer will not adapt the SCRMPs $\left(U_{i}=0\right)$ if they do not take in to count positive net benefits $(U_{i}^{*}$ ≤ 0).

The adaptation of SCRMPs could help save on the cost of medication and improve farmers’ health and the quality of the regional environment. It can be hard to differentiate between adopters and non-adopters regarding health quality. If experimental data are collected using randomization and counterfactual scenarios, it will be pretty easy to distinguish between adopters and non-adopters. So, there is no counterfactual evidence available in our cross-sectional data; the direct impact of adaptation can be measured by looking at the differences in outcomes between adopters and non-adopters. However, this may lead to inaccurate and biased estimates. The phenomenon of self-selection bias is critical in assessing the net effect of the adaptation on health costs. Let us assume a reduced form ordinary least square (OLS) equation that represents the relationship between the adaptation and performance variables as we consider the value of self-selection bias:

(3) $$Y_{ij}=\lambda Z_{ik}+\psi U_{i}+\varepsilon_{i}$$

where $Z_{ik}$ is the vector of the output variable such as the health cost for the ith farmer and $\varepsilon_{i}$ is the error term, similar to Equation (1); $Z_{ik}$ represents the independent variables’ vector and is the regression coefficient. It might be possible that the decision to adopt ($U_{i}$), which is assumed to be independent in the above equation, Equation (3), may be influenced by some unobservable factors, e.g., knowledge, perception, or farmers’ skills, which are already part of the error term $\left(\varepsilon_{i}\right)$ of Equation (3). In other words, the error term $\left(\varepsilon_{i}\right)$ of Equation (3) may be correlated with the error term $\left(\mu_{i}\right)$ of Equation (1), and the resulting selection bias may yield biased estimates [55]. There are many methods in the literature that have been adopted to overcome this problem, such as the Heckman two-step method and instrumental variables (IV) approach. In this procedure, at least one variable in the treatment equation must serve as an instrument for the determination of the outcome equation. In short, finding a valid instrument is a challenge [56]. Furthermore, the OLS and IV procedures constrain the model to have a linear functional form, meaning that the treatment and control variable coefficients are identical.

#### 2.3.2. Propensity Score Matching

We used Propensity score matching (PSM), a widely adopted approach for dealing with the issue of selection bias [57,58,59]. The impact on the overall health costs of SCRMPs on treatment (adopters) and control (non-adopters) groups was assessed by using the PSM. The PSM method consists of two stages. First, to approximate propensity score matching, the dependent variable (adoption of sustainable residue management practices and burning) was regressed against the various independent variables using logistic regression. Second, the nearest neighbor matching (NNM) was used to compare the two groups of farmers by using their propensity score determined in the first stage [60]. This method of matching treatment (adopters) and control (non-adopters) groups allowed us to exclude the impact of observable variables on the outcome variable (healthcare costs) [61].

#### 2.3.3. Sensitivity Analysis

PSM’s main goal is to stabilize the measured distribution of covariates across classes of adapters and non-adopters [62]. There’s a risk that any unexplained variables may simultaneously influence the adaptation decision and the outcome variable. Moreover, latent bias and matching estimates may cause a robustness problem [63]. As a result, after matching, we performed a series of model adequacy tests to ensure that the distribution of covariates between the two groups was consistent. For instance, available indicators such as pseudo R^2^, F-statistics, and standardized mean differences before and after matching were calculated.

## 3. Results and Discussions

### 3.1. Descriptive Statistics

The results of descriptive statistics are shown in Table 1. According to these, 51% of farmers do not burn crop residues after harvesting and instead use sustainable crop residue management practices. The results revealed that almost half of the respondents are aged between 30 and 50 years. Additionally, around 30% were 50 years old. Those farmers aged 30 years old accounted for 22% of the total sample. Likewise, half of the farmers have less than 20 years farming experience. Education wise, almost half of the respondents only received a primary education, whereas only 20% of the respondents have an elementary or high school level education. The majority of the farmers are small landholders and have land up to 4 acres. More than 32% of farmers have a farm size of between 5 and 12 acres. The farmers cultivating the land of more than 12 acres were considered to be large farmers. Around 30% of farmers have an annual income that is less than 250,000 Pak Rupees (PKR), while 33.8% earn between PKR 250,000 and 50,0000, and 43.3% earn more than PKR 500,000 and above. The results also show that farmers do not have enough income or wealth to access the necessary equipment to adopt sustainable crop waste management practices. Farmers in study areas have limited access to farm machinery. Only 24.5% of the farmers have access to tractor trollies and other farm implements such as disc ploughs, rotavators, and threshers. This study’s results align with those of [64,65], which revealed that farmers had higher ages, lower education levels, and limited availability of resources in developing countries. Further, limited access to farm machinery and tool may be an essential determinant of the non-adoption of sustainable crop residue management.

### 3.2. Health Impacts and Health Costs of Crop Residue Burning

It is acknowledged that significant crop residue burning can negatively affect local communities [66]. However, to explore these negative health impacts and associated costs, we asked households to recall what kind of health issues they or their family face during the burning of crop residues season. The significant adverse health impacts reported by farm households include coughing (45%), eye irritation (33%), headache (31%), nausea (29%), skin irritation (23%), and respiratory allergies (22%) (Table 2). Blurred vision, bronchial infection, dizziness, asthma, and fatigue are other minor side effects. Respiratory allergies are directly connected with air pollution. Further, the severity of the disease also depends on temperatures and the dispersion of plumes from burning. These results are consistent with previous reports and studies that have found a variety of negative consequences for households due to crop residue burning [67,68,69]. Cheng et al. (2011), [70] also found that agricultural crop residue burning contributes to adverse health effects in indoor and outdoor environments, such as cardiac and respiratory morbidity and mortality. Moreover, 7350–16,200 premature deaths and 6.0 million asthma attacks/year were reported in Delhi due to increased emissions from crop residue burning in the north-western part of India [71].

Further, we found that the intensity of negative impacts varies across study districts depending upon the intensity of crop residue burning. For instance, most households in Gujranwala, where rice burning is a huge problem, reported more negative impacts than other districts. Gujranwala is one of the leading cities in rice production due to easy access to water resources. Resultantly, more residual is produced. Due to ease, farmers burn the crop residue to get rid of it, contributing highly to GHG emissions. Therefore, Gujranwala is an environmental vulnerability city in Punjab district, Pakistan. Crop burning activity has been increased due to the lack of storage facilities, and market opportunities also drive the farmers to burn crop residues [72]. Table 3 demonstrates the perception of the respondents regarding crop residue burning intensity and its impact on health.

Further, we also explored other indirect or non-health-related impacts of crop residue burning (Table 4). Many respondents reported that crop residue burning directly affects their work productivity as they fall ill due to heavy smoke at the workplace. Furthermore, many farmers claimed that the smoke generated by burning caused smog and caused accidents on major highways. Farmers in Gujranwala and Rahim Yar Khan, in particular, have noticed this phenomenon. Crop residue burning has resulted in the deaths of some people in serious situations, either as a result of accidents or health problems.

### 3.3. Health Cost Due to Crop Residue Burning

Further, we estimated the economic value of health damages caused due to crop residue burning. In chronic cases, exposure to a high level of air pollution may cause permanent health injuries such as the development of lung diseases such as asthma, Chronic Obstructive Pulmonary Disease (COPD), bronchitis, lung capacity loss, and emphysema, cancer, etc. [71]. For this purpose, we acquired information about the number of visits to the hospital or clinic for each case and asked for information on different aspects of each visit, including the cost of medication, traveling/transportation, self-treatment, and preventive measures (Table 5). In the end, we calculated the total health cost for each health impact. The present study estimates health costs due to burning crop residue among households. The results revealed that the total medication cost on average for chronic diseases is USD 13.37, and for non-chronic illnesses, USD 8.79. The cost of preventive measures is very low among the farmers.

Moreover, farmers rely on self-treatment, which deteriorates the efforts to curb the burning of residues. The medication cost and traveling are also a cause of the increase in the expenses. Especially, the availability and access to specialized health facilities are not enough in Punjab’s rural areas. People also use domestic methods, which are also very cheap. However, adopting preventive measures during burning days is also not very common. Farmers are responsible for making management decisions that will optimize crop yields and minimize environmental impacts.

### 3.4. Farm Level Adoption of SCRMPs

**Figure 4:**
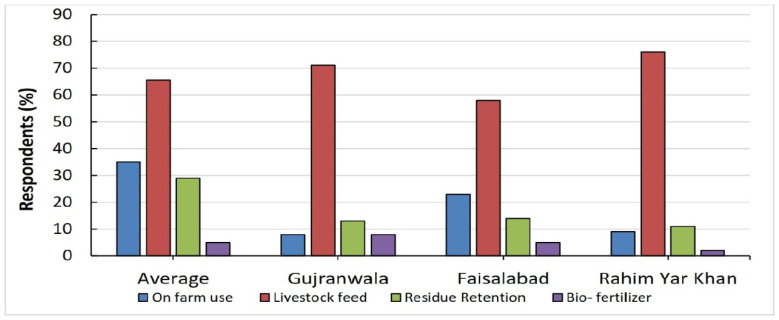
Farmers’ residue management practices in three Punjab districts.

We assessed actual adoption in the study districts to evaluate farmers’ current understanding of sustainable residue management practices and the farm-level adaptation mechanism. According to the findings, farmers in three research districts used various methods to treat crop residue (Figure 4). The decomposition of crop residues has both positive and negative impacts on crop production. Therefore, the farmer should adopt practices that have a maximum positive effect on the environment and minimum negative effect on human health and the environment. For example, soil management with crop residues covers a wide range of aspects, such as residue decomposition, soil erosion control, nutrient recycling and availability to plants, and various conservation practices related to tillage for maximizing crop yields. So, if accurate knowledge about sustainable adoption of crop management practices provides to farmers, they are willing to adopt SCRMPs. It is well known that the sustainable management of crop resides can efficiently improve the soil chemical properties, such as pH, electrical conductivity, cation exchange capacity (CEC), and the transformation of different primary and secondary plant nutrients. So, there is need to provide this important information to farmers.

The use of crop residue as livestock feed was the key sustainable crop residue strategy adopted by more than half of the farmers. The results imply that feed crop residual to livestock does not break the cycle of nutrient and biomass return to the soil since these can be returned in manure, which improves soil fertility. Therefore, sustainable livestock feed is necessary for optimal use of crop residue. Other measures include on-farm use, residue retention, and bio-fertilization. In the study area, most farmers are smallholders and often rear livestock to manage their livelihood. Therefore, they often use crop residue as a source of animal feed. For this purpose, they collect and store residue in one place. Mostly, farmers keep residue in the open field due to the non-availability of a warehouse or appropriate storage place.

Further, farmers often use crop residue for bedding and shelter for their livestock. It is done mainly in winter to give comfort to milking animals. Farmers reported that this practice has resulted in a positive impact on the quality and quantity of milk. Furthermore, some farmers in the study districts use the residue retention technique. Farmers in Gujranwala, for example, have implemented a residue retention policy to use crop residues to enhance soil and water quality. This strategy would be beneficial in terms of agronomical and economically. These factors would lead to significant advantages (i.e., reducing costs of fertilizers and water). Makkar (2016) [73] explored that using crop residue for livestock feed would help to improve the three traditional sustainability pillars (economic, environmental and social). Similarly, [74,75,76] also reported that adopting the residue retention technique and conservation agriculture improves soil health nutrients ratio and increases water conservation in the field.

**Figure 5:**
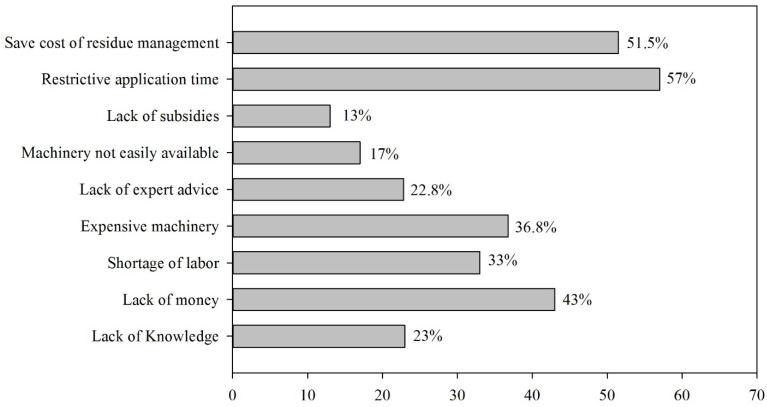
Constraints faced by the farmers.

In addition to positively impacting sustainable residue management practices, the adoption rate is still very low due to various constraints, even though sustainable crop residual management improves the fertility and productivity of the soil. We further explored farmers’ key hurdles and limitations restricting them from adopting sustainable crop residue management practices. According to the study findings, the saving cost of residue management, restrictive application time, limited knowledge, lack of training in advanced technologies, and limited financial capacity are critical reasons for low adoption (Figure 5). Many other studies, e.g., [76,77], have also identified the current policies, financial constraints, etc., low education level restricts farmers from adopting these practices. So results imply that landholding should increase to achieve the optimal benefits from sustainable crop residue management. Small landholding farmers should have proper access to credit and fertilizer facilities. Education and lack of proper training are the main barriers to optimal crop residue use.

**Figure 6:**
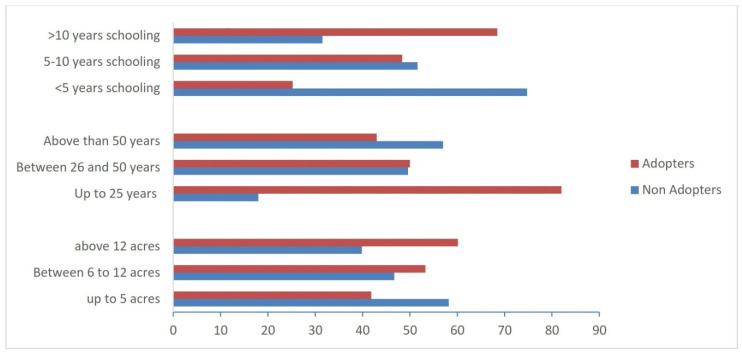
Relationship between adaptation and socio-demographic properties.

We also examined how crop residue management practices were adopted by different farmers based on their level of education, age, and landholding scale (see Figure 6). Farmers were classified into three groups based on their level of education: (1) farmers who are illiterate or have had less than 5 years of schooling; (2) farmers who have had 5 to 10 years of schooling; and (3) farmers who have had more than 10 years of schooling. Farmers were also classified into three age groups: (1) those under the age of 25, (2) those between the ages of 26 and 50, and (3) those over 50. Farmers were classified into three groups based on the size of their farms: (1) small-scale farmers with up to 5 acres of land, (2) medium-scale farmers with 6 to 12 acres of land, and (3) large-scale farmers with more than 12 acres of land.

The results in Figure 6 show a positive association of adoption decisions with education level. About 68% of the farmers with higher education do not burn their crop residues and use sustainable residue management practices. Of the farmers with 5–10 years of education, only 48% adopted other practices. Similar positive associations of this behavior with education level were reported by [74,78]. Furthermore, Figure 6 revealed that farmers with a higher education adopted these practices than less educated farmers. Likewise, the adoption rate among the young farmers is also high compared to the old farmers. These findings are consistent with the findings of [78,79,80], who found a positive relationship between education level and sustainable crop residue use. The proportion of adoption among young and middle-aged farmers is around 82–50% compared to old-age farmers. The share of farmers with landholding up to 5 acres is 42%, but the large-scale farmer’s rate was almost 60%. These findings indicate that large-scale farmers were less constrained in implementing residue management practices. These findings are consistent with previous research, e.g., [78,80], which found a connection between landholding and the adoption of sustainable residue management practices.

### 3.5. Knowledge, Understanding, and Sustainable Residue Management Activities

In the next step, we explored the level of awareness and knowledge about the hazardous impact of crop residue burning among adopters and non-adopters. We found that the level of knowledge and awareness was higher in the case of adopters as compared to non-adopters. This implies that awareness about negative impacts plays an important role in deciding between the adoption of crop residue practices. Furthermore, we discovered that farmers who were aware of various alternative or beneficial aspects of sustainable crop residue management practices were more likely to follow them.

Knowledge of the hazardous impact of burning crop residue on health was explored. Further, we asked about the households who suffered any illness because of the residue burning. We categorized the farmers into two categories, those who do not burn the crop residues are the adopters, and the others are non-adopters. Mostly, non-adopters know that burning cause hazardous health impact. Likewise, burning also induced illness among households during the harvesting season (Table 6). The number of adopters is higher than non-adopters. This result implies the lack of information about damages associated with crop residue burning. Most adopters know about sustainable crop reside management, such as livestock feed, reside used as a shelter for livestock, etc., so they optimally use it rather than burning it. The results can also be related to their financial condition. They are holding small land, so the residue produced is used for livestock to avoid the extra cost of feeding their livestock.

### 3.6. Empirical Results

#### 3.6.1. Empirical Results of the Propensity Score Matching

To estimate propensity scores, logistic regression is used to regress the likelihood of implementing residue management activities against a variety of covariates. Table 7 displays the effects of the propensity score estimate.

We analyzed the farm-level adaptation measures across different categories of farmers, i.e., the farm size and their educational level. The farm size of adapters also positively impacts farmers’ adaptation for residual crop management. The results are consistent with those of [81,82]. Education has been identified as an important component of implementing sustainable agricultural residue management strategies [83,84]. The results imply that an increase in education increases the likelihood of implementing sustainable crop residue management practices. Educated farmers adapted more compared to less educated farmers. These results are consistent with other research findings, confirming that the number of years of schooling plays a vital role in pushing humans to make economically sustainable and environmentally health-conscious decisions [85,86].

We found inconclusive results regarding the relationship between age and the adoption of new practices among farmers. The variable for the farm household’s age, on the other hand, is negatively but insignificantly correlated with adoption decisions. This finding may be attributed to the predicted opposite impact of age on the likelihood of implementing management activities that operate across the decision-networks maker’s and planning horizon [77,87]. The results may imply that people with a higher age bracket are less indulged in adoption practices. Similarly, income has no significant impact on farmers’ decisions to follow sustainable crop residue management practices.

Tube well ownership does significantly affect the farmer’s decision to adopt sustainable residue management practices. This is true because the easy availability of water at farms makes farmers flexible in making decisions on harvesting and sowing crops. This flexibility also allows them to manage harvest and crop residue effectively and easily.

The rotavator ownership reduces the probability of adoption, possibly reflecting that those households used this instrument for commercial purposes and less need to use it for their purpose. Further, variables related to farm machinery and implements such as tractor trollies and disc ploughs are significantly related to the adoption decision, implying that farmers having easy access to farm machinery can easily adjust their sowing and harvesting timing, allowing them to manage their crop residues in a better way.

Furthermore, having a tractor trolley is a key component in using agricultural residue responsibly, as farmers sometimes have difficulty transferring their residue to other locations. As a result of the lack of adequate transportation or cost of the transportation, they may be influenced to burn crop residue as a convenient and cost-effective technique. The results are in line with other study, e.g., [64].

The results imply that access to extension services and canal water information may positively impact the farmers’ adaptation decision. Access to extension services and canal water information has positive coefficients and tends to expedite anti-burning sustainable management practices. These findings proved our assumption before the analysis and aligned with the other studies’ results, e.g., [72,88].

In the case of residue management activities, however, the negative coefficient of the weather forecasting variable indicates that farmers could not relate their decision behavior to the weather situation. This result also depicts the poor weather forecast facilities available to the farmer. This region is not rich in technological innovations; therefore, farmers cannot adopt residual management practices. The results also imply that most of the adapters use weather forecasting information from different sources to adjust management options, which may not be accurate due to differences in weather conditions in these three regions. Each region has different weather conditions, negatively impacting overall crop residual management. These findings are not consistent with the results of other studies, e.g., [81,87,89].

The results for regional dummies are partially consistent with a previous study [51,90,91]. Furthermore, the negative coefficients for regional dummies imply that farmers in both districts (Gujranwala and Faisalabad) were less likely to adapt residual waste management practices than the Rahim Yar Khan district farmers. This may be because farmers in Rahim Yar Khan are more concerned with health and the environment or do not have any awareness/knowledge about the adverse effects of residual burning.

#### 3.6.2. Impact of Sustainable Crop Residue Management Practices on Health Costs

It is understood that adopting sustainable crop residue management practices may generally reduce health costs in the areas where farmers do not burn crop residues. We calculated the casual impact of adopting sustainable residue management practices on health costs to test this hypothesis. For this purpose, first propensity scores were calculated using logistic regression. After calculating the propensity scores based on identical propensity scores, the nearest neighbor matching approach was used to align adopters (treatment group) and non-adopters (control group). The nearest neighboring method (NNM) discarded non-adopters during the matching process, resulting in a substantial reduction in the total sample size from 420 to 214 for post-matching effect analysis.

The post-matching results are presented in Table 8, which reveals that the adoption of sustainable crop residue management practices significantly reduces the health cost. Here, the ATE shows the Average Treatment Effect (ATE) for all without matching and correcting the biases. In contrast, ATT shows the average treatment effect for treated, i.e., the impact after matching and correcting the biases. The results showed that the overall impact of sustainable crop residue management practices (ATE) without correcting biases was more (PKR 897) than the impact calculated after correcting the biases. The value of ATT (PKR 312) shows that adopting sustainable crop residue management practices will reduce the health cost by PKR 312 per season.

Further, the results of sensitivity analysis presented in Table 9 also confirm the adequacy of our results. The results show a decline in R-square value, F-value, and mean standard differences, indicating reduced biases. Overall, more than 55% of business has been reduced through the matching process. The impact of health costs is only determined by the adoption of sustainable crop residue management practices.

## 4. Conclusions

Agricultural crop residue burning has emerged as an essential challenge in the agricultural production system because of rising air pollution episodes, the release of short-lived climate pollutants, and declining soil health. Crop residual burning negatively affects the health and environment of rural livelihoods in Pakistan adversely. Thus, timely adaptation is desirable to reduce potential losses at the farm level. This case study analyzed farmers from rural Pakistan and provided insights into their adaptation to residual management determinants. This study reveals the extent to which farmers perceive residue as a problem and adapt their residual management practices accordingly.

The results of our study revealed that adopting sustainable crop residue management is considered the best alternative to prevent the burning’s hazardous impact on the ecosystem. However, farmers end up with higher costs in terms of medication expenses due to facing health issues because of the burning. Likewise, a loss of working hours and medication costs are significant contributors to the losses.

The cost reduction shows the effective approach to tackle the expenses. Moreover, results also revealed that adopting sustainable crop residue management practices helped reduce the expenses due to health issues. This study also confirms that sustainable utilization of crop residues can improve households’ health status and help reduce the financial burden. Overall, this study proved and reckons that the sustainable utilization of crop residue is beneficial in curbing the hazardous impact of residue burning on the environment. However, farmers are still not well aware of these benefits due to the numerous constraints.

This implies that large-scale awareness campaigns in both rural and urban areas should be created. Government agencies’ positions are crucial to this campaign’s success. Focus group awareness, workshops, and community mobilization activities are highly feasible choices. Similarly, capacity building of extension workers and local stakeholders could also help enhance the sustainable management of crop residues. Research institutes, government agencies, non-governmental organizations (NGOs), and the private sector can significantly remove these constraints through the vibrant collaboration for capacity building and dissemination of innovative practices among farmers.

Furthermore, policies should be updated based on on-the-ground research and small farmers, who account for more than two-thirds of Pakistan’s total farmer population. Both of these measures will help to alleviate the negative effects of climate change and can aid farmers in improving their well-being and ensuring better health. In addition, they will help to minimize the negative impacts of crop residue burning. In this regard, the interventions from the circular bioeconomy involve the recirculation of material flows and the adoption of the restoration cycle.

This study used cross sectional data to explore the nexuses between the positive aspects of adopting SCRMPs and their impact on human health. Nevertheless, there are numerous limitations worth mentioning. First, this study concerns the elicitation responses related to health impacts and burning instead of scientific data. Secondly, research is necessary for improvements and was validated with satellite-based fire count data. Furthermore, it can be extended by estimating the GHGs emission load due to crop residue burning activity over said regions during the burning months to assess its impact on health. It also helps to widen the scope of the study and make it more comprehensive that encompassing the problem well.

## Figures and Tables

**Table 1:** Descriptive statistics.

Variables	Description	Percentage (%)	
Age	Up to 30 years	21.9	
	31–50	47.9	
	51–70	28.8	
	71 and above	1.4	
Education level	Up to the Primary education	44.3	
	Elementary School	20.5	
	High School	23.6	
	College or above	11.7	
Farm size	Up to 4 acres	38.3	
	5–12 acres	32	
	13–24 acres	15.2	
	More than 24 acres	14.5	
Annual income	Up to PKR 250,000	22.9	
	PKR 250,000–500,000	33.8	
	PKR 500,000 and above	43.3	
**(Total number of sample size n = 420)**		**Yes (%)**	**No (%)**
Adoption	Dummy takes the value 1 if do not burn and 0, otherwise	50.8	48.9
Tube well ownership	Dummy takes the value 1 if own tube well and 0, otherwise	44	66
Mobile	Dummy takes the value 1 if own mobile and 0, otherwise	97	3
Tractor Trolley	Dummy takes the value 1 if own Tractor trolley and 0, otherwise	24.5	75.5
Rotavator	Dummy takes the value 1 if own rotavator and 0, otherwise	29.5	70.5
Extension	Dummy takes the value 1 if use extension service and 0, otherwise	62.5	37.5
Credit	Dummy takes the value 1 if use credit and 0, otherwise	67.5	32.5
Thresher	Dummy takes the value 1 if have Thresher and 0, otherwise	12.6	87.4
Weather forecast	Dummy takes the value 1 if check weather forecast and 0, otherwise	63.33	36.66
Canal water information	Dummy takes the value 1 if collect canal water information and 0, otherwise	46.6	53.4
Member of Farmers organization	Dummy takes the value 1 if member of FO and 0, otherwise	5	95
Disc Plough	Dummy takes the value 1 if own Disc plough and 0, otherwise	42.6	57.3
Gujranwala	Dummy takes the value 1 if from Gujranwala and 0, otherwise	33	67
Faisalabad	Dummy takes the value 1 if from Faisalabad and 0, otherwise	33	67
Rahim Yar Khan	Dummy takes the value 1 if from Faisalabad and 0, otherwise	33	67

**Table 2:** Health damages due to crop residue burning (percentage response of respondents).

Health Impacts	Gujranwala	Faisalabad	R. Y. Khan	Overall
Coughing	49.6	40.3	45	45
Eye irritation	42	33	24.5	33
Headache	32	28	32.9	31
Nausea	35	21	32	29
Skin irritation	27.6	19	23.3	23
Respiratory Allergies	37.6	12	17.1	22
Blurred vision	20	12.3	18.5	17
Dizziness	11	7.1	15	11
Asthma	11	13.9	5.2	10
Bronchial Infection	14.1	10.1	6	10

Some respondents reported more than one health impact, so the percentage would not be equal to 100.

**Table 3:** Relating the intensity of crop residue burning with the intensity of suffering (percentage response of respondents’ perceptions).

Districts	Do You Think the Intensity of Crop Residue Burning Increase (Yes)	Do You Think Health Impact Increase Due to Crop Residue Burning (Yes)
Gujranwala	56%	69%
Faisalabad	41.6%	55%
Rahim Yar Khan	54.3%	62%

**Table 4:** Other issues associated with crop residue burning. (percentage).

Districts	Loss of Work Productivity for Working Members	Having Observed Accident Happening Due to Smoke	No. of Injuries/Deaths Due to Smoke
Gujranwala	31	23	27
Faisalabad	23	14	13
Rahim Yar Khan	35	34	22

**Table 5:** Health costs incurred caused by crop residue burning (USD/season).

District Name	Traveling/ Transportation	Self-Treatment	Preventive Measure	Medication (Doctor, Hospital Charges/Medicine Cost)	
				Chronic Diseases	Non-Chronic Diseases
Gujranwala	1.85	0.73	0.85	13.21	9.63
Faisalabad	2.13	1.52	0.75	12.87	8.32
RYK	2.27	1.32	0.67	14.02	8.43
Total	2.08	1.20	0.76	13.37	8.79

**Table 6:** Knowledge about the hazardous impact of crop residue burning (percentage).

Particulars	Adopters	Non-Adopters
Are you aware of the harmful impact of crop residue burning on health?		
Yes	55.07	75.58
No	44.93	24.42
Are you aware of different alternative crop residue management practices?		
Yes	59.42	58.68
No	40.57	41.31

**Table 7:** Estimation of the propensity score matching through logistic regression.

Variables	Estimator	Standard Deviation	Z-Value
Education	0.17	0.031	5.56 ***
Age	−0.01	0.009	−1.12
Income	5.89	0.001	0.11
Farm size	0.04	0.01	2.44 **
Tube well ownership	0.39	0.15	2.63 ***
Tractor Trolley	1.13	0.47	2.39 **
Rotavator	−1.65	0.55	−3.01 ***
Disc Plough	1.84	0.34	5.43 ***
Thresher	0.24	0.58	0.43
Distance output market	0.01	0.01	0.89
Paved Road	−0.07	0.05	−1.27
Member of Farmers organization	0.991	0.64	1.54
Extension Services	0.65	0.32	1.98 **
Weather forecast	−1.45	0.35	−4.14 ***
Canal water information	0.55	0.30	1.83 *
Credit	0.10	0.28	0.36
Mobile	0.80	1.01	0.79
Gujranwala	−1.21	0.34	−3.51 ***
Faisalabad	−0.56	0.31	−1.79 *
Rahim Yar Khan	Omitted		
Number of observations (420), LR chi^2^ (19) (174.30), Prob > chi^2^ (0.0000), Log likelihood (−203.89), Pseudo R^2^ (0.2994)			

*** *p* < 0.01, ** *p* < 0.05, * *p* < 0.1.

**Table 8:** Impact of adoption on health costs.

Outcome	ATT	ATE	No. of Treated	No. of Control
Health Cost	−312 **	−897 **	107	107

** 5% level of significance.

**Table 9:** Impact of balancing covariates before and after matching.

Indicators of Covariates Balancing	Before Matching	After Matching
Pseudo R^2^	0.2994	0.0721
*p*-value Log likelihood	0.001	0.23
F-stat	79.22	10.44
Mean standardized difference	0.26	0.03
Total% bias reduction (%)	-	56

## Data Availability

Data published in this study are available on request from the corresponding author. The data are not publicly available due to the policy of the research project.

## References

[1] Bruinsma J. (2003). World Agriculture: Towards 2015/2030: An FAO Perspective.

[2] FAO (2014). The State of Food Insecurity in the World: Strengthening the Enabling Environment for Food Security and Nutrition.

[3] Taheri F., Azadi H., D’Haese M. (2017). A World without Hunger: Organic or GM Crops?. Sustainability.

[4] Mendola M. (2008). Migration and technological change in rural households: Complements or substitutes?. J. Dev. Econ..

[5] Akudugu M.A., Guo E., Dadzie S.K. (2012). Adoption of modern agricultural production technologies by farm households in Ghana: What factors influence their decisions. J. Biol. Agric. Healthc..

[6] Chen X.P., Cui Z.L., Fan M.S., Vitousek P., Zhao M., Ma W.Q., Wang Z.L., Zhang W.J., Yan X.Y., Yang J.C. (2014). Producing more grain with lower environmental costs. Nature.

[7] Van Keulen H. (2007). Quantitative analyses of natural resource management options at different scales. Agric. Syst..

[8] Qin L., Wang Y., Wu Y., Wang Q., Luo L. (2015). Assessment of nitrate leakage and N 2 O emission from five environmental-friendly agricultural practices using fuzzy logic method and empirical formula. Environ. Monit. Assess..

[9] Jallow M.F.A., Awadh D.G., Albaho M.S., Devi V.Y., Thomas B.M. (2017). Pesticide risk behaviors and factors influencing pesticide use among farmers in Kuwait. Sci. Total Environ..

[10] Zhang H.F., Hu J., Qi Y.X., Li C.L., Chen J.M., Wang X.M., He J.W., Wang S.X., Hao J.M., Zhang L.L. (2017). Emission characterization, environmental impact, and control measure of PM2.5 emitted from agricultural crop residue burning in China. J. Clean. Prod..

[11] Shukla S.K., Singh K.K., Pathak A.D., Jaiswal V.P., Solomon S. (2017). Crop Diversification Options Involving Pulses and Sugarcane for Improving Crop Productivity, Nutritional Security and Sustainability in India. Sugar Tech.

[12] Sun J., Peng H., Chen J., Wang X., Wei M., Li W., Yang L., Zhang Q., Wang W., Mellouki A. (2016). An estimation of CO_2_ emission via agricultural crop residue open field burning in China from 1996 to 2013. J. Clean. Prod..

[13] Faisal M., Abbas A., Cai Y., Ali A., Shahzad M.A., Akhtar S., Raza M.H., Ajmal M.A., Xia C., Sattar S.A. (2021). Perceptions, Vulnerability and Adaptation Strategies for Mitigating Climate Change Effects among Small Livestock Herders in Punjab, Pakistan. Int. J. Environ. Res. Public Health.

[14] Busch J. (2015). Climate Change and Development in Three Charts|Center For Global Development. http://www.cgdev.org/blog/climate-change-anddevelopment-three-charts,.

[15] UNEP (2009). Hoff a Major Scientific Study on black Carbon Inter-Comparison.

[16] PBS (2017). Provisional Summary results of 6TH Population and Housing Census-2017|Pakistan Bureau of Statistics.

[17] Khokhar M.F., Khalid T., Yasmin N., De Smedt I. (2015). Spatio-temporal analyses of formaldehyde over Pakistan by using SCIAMACHY and GOME-2 observations. Aerosol Air Qual. Res..

[18] Ahmed T., Ahmad B. (2014). Burning of crop residue and its potential for electricity generation. Pak. Dev. Rev..

[19] Bakhsh K., Rauf S., Zulfiqar F. (2018). Adaptation strategies for minimizing heat wave induced morbidity and its determinants. Sustain. Cities Soc..

[20] Barbier B., Yacouba H., Karambiri H., Zorome M., Some B. (2009). Human Vulnerability to Climate Variability in the Sahel: Farmers’ Adaptation Strategies in Northern Burkina Faso. Environ. Manag..

[21] Raza M.H., Abid M., Yan T., Ali Naqvi S.A., Akhtar S., Faisal M. (2019). Understanding farmers’ intentions to adopt sustainable crop residue management practices: A structural equation modeling approach. J. Clean. Prod..

[22] Riaz R., Hamid K. (2018). Existing Smog in Lahore, Pakistan: An Alarming Public Health Concern. Cureus.

[23] Glum J. (2015). Pakistan heat wave 2015: Death toll exceeds 1200 as Karachi struggles with continued extreme weather during Ramadan. International Business Times.

[24] Reporter S. (2016). Burning of leftover crop by farmers caused smog in Punjab. Pakistan Today.

[25] Mukhtar F. (2017). The rising menace of Smog: Time to act now. J. Ayub Med. Coll. Abbottabad.

[26] Zhao H., Zhang X., Zhang S., Chen W., Tong D.Q., Xiu A. (2017). Effects of agricultural biomass burning on regional haze in China: A review. Atmosphere.

[27] Abbas A., Amjath-Babu T.S., Kächele H., Müller K. (2015). Non-structural flood risk mitigation under developing country conditions: An analysis on the determinants of willingness to pay for flood insurance in rural Pakistan. Nat. Hazards.

[28] Thumaty K.C., Rodda S.R., Singhal J., Gopalakrishnan R., Jha C.S., Parsi G.D., Dadhwal V.K. (2015). Spatio-temporal characterization of agriculture residue burning in Punjab and Haryana, India, using MODIS and Suomi NPP VIIRS data. Curr. Sci..

[29] Lima I.M., White M. (2017). Sugarcane bagasse and leaf residue biochars as soil amendment for increased sugar and cane yields. Int. Sugar J..

[30] Jiang L., Zhang J., Wang H.H., Zhang L., He K. (2018). The impact of psychological factors on farmers’ intentions to reuse agricultural biomass waste for carbon emission abatement. J. Clean. Prod..

[31] Smith P., Martino D., Cai Z., Gwary D., Janzen H., Kumar P., McCarl B., Ogle S., O’Mara F., Rice C. (2007). Policy and technological constraints to implementation of greenhouse gas mitigation options in agriculture. Agric. Ecosyst. Environ..

[32] Hasan E. (2013). Proposing mitigation strategies for reducing the impact of rice cultivation on climate change in Egypt. Water Sci..

[33] Domingo J., De Miguel E., Hurtado B., Métayer N., Bamière L., Pardon L., Bochu J., Pointereau P., Pellerin S. (2014). Measures at farm level to reduce greenhouse gas emissions from EU agriculture. Notes. Policy Dep. B Struct. Cohes. Policies..

[34] Sheikh A.D., Rehman T., Yates C.M. (2003). Logit models for identifying the factors that influence the uptake of new ‘no-tillage’technologies by farmers in the rice–wheat and the cotton–wheat farming systems of Pakistan’s Punjab. Agric. Syst..

[35] Sarwar M.N., Goheer M.A. Adoption and impact of zero tillage technology for wheat in rice-wheat system—Water and cost saving technology. A case study from Pakistan (Punjab). Proceedings of the International Forum on Water Environmental Governance in Asia; Citeseer.

[36] Abid M., Schilling J., Scheffran J., Zulfiqar F. (2016). Climate change vulnerability, adaptation and risk perceptions at farm level in Punjab, Pakistan. Sci. Total Environ..

[37] Ali A., Erenstein O. (2017). Assessing farmer use of climate change adaptation practices and impacts on food security and poverty in Pakistan. Clim. Risk Manag..

[38] Haider M.Z. (2013). Determinants of rice residue burning in the field. J. Environ. Manag..

[39] Ahmed T., Ahmad B., Ahmad W. (2015). Why do farmers burn rice residue? Examining farmers’ choices in Punjab, Pakistan. Land Use Policy.

[40] Mendoza T.C. (2015). Enhancing Crop Residues Recycling in the Philippine Landscape. Environmental Implications of Recycling and Recycled Products.

[41] Jiang W., Yan T., Chen B. (2021). Impact of media channels and social interactions on the adoption of straw return by Chinese farmers. Sci. Total Environ..

[42] Gai H., Yan T., Zhang A., Batchelor W.D., Tian Y., Gai C., Yan H., Zhang T., Batchelor A., Tian W.D. (2021). Exploring Factors Influencing Farmers’ Continuance Intention to Crop Residue Retention: Evidence from Rural China. Int. J. Environ. Res. Public Health.

[43] Akhtar S., Gu-cheng L.I., Ullah R., Nazir A., Iqbal M.A., Raza H., Iqbal N., Faisal M. (2017). Factors influencing hybrid maize farmers’ risk attitudes and their perceptions in Punjab Province, Pakistan. J. Integr. Agric..

[44] (2013). BoS Punjab Development Statistics, Bureau of Statistics 2013.

[45] Ahmed U.I., Ying L., Bashir M.K., Abid M., Zulfiqar F. (2017). Status and determinants of small farming households’ food security and role of market access in enhancing food security in rural Pakistan. PLoS ONE.

[46] Chaudhry Q.Z., Rasul G. (2004). Agroclimatic classification of Pakistan. Sci. Vis..

[47] Ghafoor A., ur Rehman T., Munir A., Ahmad M., Iqbal M. (2016). Current status and overview of renewable energy potential in Pakistan for continuous energy sustainability. Renew. Sustain. Energy Rev..

[48] Irfan M., Riaz M., Arif M.S., Shahzad S.M., Saleem F., van den Berg L., Abbas F. (2014). Estimation and characterization of gaseous pollutant emissions from agricultural crop residue combustion in industrial and household sectors of Pakistan. Atmos. Environ..

[49] Singh R.P., Kaskaoutis D.G. (2014). Crop residue burning: A threat to South Asian air quality. Eos Trans. Am. Geophys. Union.

[50] Faisal M., Abbas A., Xia C., Haseeb Raza M., Akhtar S., Arslan Ajmal M., Mushtaq Z., Yi C. (2021). Assessing small livestock herders’ adaptation to climate variability and its impact on livestock losses and poverty. Clim. Risk Manag..

[51] Abid M., Schneider U.A., Scheffran J. (2016). Adaptation to climate change and its impacts on food productivity and crop income: Perspectives of farmers in rural Pakistan. J. Rural Stud..

[52] Hauswirth D., Pham T.S., Wery J., Tittonell P., Jourdain D., Affholder F. (2015). Exploiting farm typologies for designing conservation agriculture systems: A case study in northern Vietnam. Cah. Agric..

[53] Tittonell P.A. (2007). Msimu wa Kupanda: Targeting Resources within Diverse, Heterogeneous and Dynamic Farming Systems of East Africa. Ph.D. Thesis.

[54] Irfan M., Riaz M., Arif M.S., Shahzad S.M., Hussain S., Akhtar M.J., van den Berg L., Abbas F. (2015). Spatial distribution of pollutant emissions from crop residue burning in the Punjab and Sindh provinces of Pakistan: Uncertainties and challenges. Environ. Sci. Pollut. Res..

[55] Kato E., Ringler C., Yesuf M., Bryan E. (2011). Soil and water conservation technologies: A buffer against production risk in the face of climate change? Insights from the Nile basin in Ethiopia. Agric. Econ..

[56] Thoemmes F. (2012). Propensity score matching in SPSS. arXiv.

[57] Elahi E., Abid M., Zhang L., ul Haq S., Sahito J.G.M. (2018). Agricultural advisory and financial services; farm level access, outreach and impact in a mixed cropping district of Punjab, Pakistan. Land Use Policy.

[58] Heckman J., Ichimura H., Smith J., Todd P. (1998). Characterizing Selection Bias Using Experimental Data. Econometrica.

[59] Ali A., Rahut D.B., Mottaleb K.A. (2018). Improved water-management practices and their impact on food security and poverty: Empirical evidence from rural Pakistan. Water Policy.

[60] Newton H.J., Baum C.F., Beck N., Cameron A.C., Epstein D., Hardin J., Jann B., Jenkins S., Kohler U. (2010). Two-part model. Stata J..

[61] Ali A., Abdulai A. (2010). The adoption of genetically modified cotton and poverty reduction in Pakistan. J. Agric. Econ..

[62] Imai K., Ratkovic M. (2014). Covariate balancing propensity score. J. R. Stat. Soc. Ser. B Stat. Methodol..

[63] Rosenbaum P.R. (2002). Observational Studies.

[64] Faisal M., Chunping X., Abbas A., Raza M.H., Akhtar S., Ajmal M.A., Ali A. (2021). Do risk perceptions and constraints influence the adoption of climate change practices among small livestock herders in Punjab, Pakistan?. Environ. Sci. Pollut. Res..

[65] Mueller V., Gray C., Kosec K. (2014). Heat stress increases long-term human migration in rural Pakistan. Nat. Clim. Change.

[66] Shyamsundar P., Springer N.P., Tallis H., Polasky S., Jat M.L., Sidhu H.S., Krishnapriya P.P., Skiba N., Ginn W., Ahuja V. (2019). Fields on fire: Alternatives to crop residue burning in India. Science.

[67] Chen J., Li C., Ristovski Z., Milic A., Gu Y., Islam M.S., Wang S., Hao J., Zhang H., He C. (2017). A review of biomass burning: Emissions and impacts on air quality, health and climate in China. Sci. Total Environ..

[68] Eskeland G., Sanchez M.J., Aranda C. (1999). Air Pollution and Mortality: Results from Santiago, Chile.

[69] Sigsgaard T., Forsberg B., Annesi-Maesano I., Blomberg A., Bølling A., Boman C., Bønløkke J., Brauer M., Bruce N., Héroux M.E. (2015). Health impacts of anthropogenic biomass burning in the developed world. Eur. Respir. J..

[70] Cheng K., Pan G., Smith P., Luo T., Li L., Zheng J., Zhang X., Han X., Yan M. (2011). Carbon footprint of China’s crop production—An estimation using agro-statistics data over 1993–2007. Agric. Ecosyst. Environ..

[71] Ghosh P., Sharma S., Khanna I. Scoping Study for South Asia Air Pollution. www.teriin.org.

[72] Lohan S.K., Jat H.S., Yadav A.K., Sidhu H.S., Jat M.L., Choudhary M., Peter J.K., Sharma P.C. (2018). Burning issues of paddy residue management in north-west states of India. Renew. Sustain. Energy Rev..

[73] Makkar H.P.S. (2016). Smart livestock feeding strategies for harvesting triple gain—The desired outcomes in planet, people and profit dimensions: A developing country perspective. Anim. Prod. Sci..

[74] Akteruzzaman M., Zaman M. (2013). Utilization Pattern of Crop Residues at Farm Level: Evidence from Diversified Rice-Based Cropping Systems in Bangladesh. Progress. Agric..

[75] Nawaz A., Farooq M., Lal R., Rehman A., Hussain T., Nadeem A. (2017). Influence of Sesbania Brown Manuring and Rice Residue Mulch on Soil Health, Weeds and System Productivity of Conservation Rice-Wheat Systems. L. Degrad. Dev..

[76] Nawaz A., Lal R., Shrestha R.K., Farooq M. (2017). Mulching Affects Soil Properties and Greenhouse Gas Emissions under Long-Term No-Till and Plough-Till Systems in Alfisol of Central Ohio. L. Degrad. Dev..

[77] Fuentes L.R., Palma A.E., Jara-Rojas R. (2012). Factors influencing the adoption of soil conservation technologies in the rainfed area of Central Chile. Rev. La Fac. Ciencias Agrar..

[78] Kumar P., Kumar S., Joshi L. (2015). Socioeconomic and Environmental Implications of Agricultural Residue Burning: A Case Study of Punjab, India.

[79] Sanz-Cobena A., Lassaletta L., Aguilera E., del Prado A., Garnier J., Billen G., Iglesias A., Sanchez B., Guardia G., Abalos D. (2017). Strategies for greenhouse gas emissions mitigation in Mediterranean agriculture: A review. Agric. Ecosyst. Environ..

[80] Shehrawat P.S., Nitu S., Parmila D. (2015). Agricultural waste awareness and utilization for healthy environment and sustainable livelihood. Sci. Pap. Ser. Econ. Eng. Agric. Rural Dev..

[81] Pali P.N., Delve R.J., White D. (2004). The adoption potential of biomass transfer and improved fallow practices in Eastern Uganda: Determining profitable and feasible options from a farmer perspective. Uganda J. Agric. Sci..

[82] Wood S.A., Jina A.S., Jain M., Kristjanson P., DeFries R.S. (2014). Smallholder farmer cropping decisions related to climate variability across multiple regions. Glob. Environ. Chang..

[83] Mugwe J., Mugendi D., Mucheru-Muna M., Merckx R., Chianu J., Vanlauwe B. (2009). Determinants of the decision to adopt integrated soil fertility management practices by smallholder farmers in the central highlands of Kenya. Exp. Agric..

[84] Bryan E., Ringler C., Okoba B., Roncoli C., Silvestri S., Herrero M. (2013). Adapting agriculture to climate change in Kenya: Household strategies and determinants. J. Environ. Manag..

[85] Abid M., Scheffran J., Schneider U.A., Ashfaq M. (2015). Farmers’ perceptions of and adaptation strategies to climate change and their determinants: The case of Punjab province, Pakistan. Earth Syst. Dyn..

[86] Antwi-Agyei P., Stringer L.C., Dougill A.J. (2014). Livelihood adaptations to climate variability: Insights from farming households in Ghana. Reg. Environ. Chang..

[87] Ashraf S., Khan G.A., Ali S., Iftikhar M. (2015). Socio-economic determinants of the awareness and adoption of citrus production practices in Pakistan. Ciência Rural.

[88] Jaleta M., Kassie M., Shiferaw B. (2013). Tradeoffs in crop residue utilization in mixed crop-livestock systems and implications for conservation agriculture. Agric. Syst..

[89] Bhuvaneshwari S., Hettiarachchi H., Meegoda J.N. (2019). Crop residue burning in India: Policy challenges and potential solutions. Int. J. Environ. Res. Public Health.

[90] Porichha G.K., Hu Y., Rao K.T.V., Xu C.C. (2021). Crop Residue Management in India: Stubble Burning vs. Other Utilizations including Bioenergy. Energies.

[91] Faisal M., Chunping X., Akhtar S., Raza M.H., Khan M.T.I., Ajmal M.A. (2020). Modeling smallholder livestock herders’ intentions to adopt climate smart practices: An extended theory of planned behavior. Environ. Sci. Pollut. Res..

